# Unusual intravesical findings: a case report on foreign body in the bladder of an adolescent female

**DOI:** 10.1093/jscr/rjae819

**Published:** 2024-12-26

**Authors:** Kholoud Alabassi, Yaser M Ata, Noora Alshahwani, Abdelrahman Elkadhi

**Affiliations:** Urology Department, Hamad Medical Corporation, Doha, Qatar; Urology Department, Hamad Medical Corporation, Doha, Qatar; Pediatric Surgery Department, Sidra Medicine, Doha, Qatar; Pediatric Urology Department, Sidra Medicine, Doha, Qatar

**Keywords:** bladder foreign body, pediatric urology, cystoscopy, lead pencil, case report, bladder perforation

## Abstract

Bladder foreign bodies (BFB) are uncommon in the pediatric population. They typically arise from self-insertion, iatrogenic factors, or trauma. Cystoscopy is the preferred intervention. A 16-year-old female presented with a [2-]day history of dysuria, suprapubic pain, and a palpable rectal foreign body. Imaging revealed an 8.2 cm radiopaque object in the bladder. Diagnostic laparoscopy confirmed no perforation. Cystoscopy identified and removed a lead pencil from the bladder. A small bladder perforation was noted post-removal. The patient had a smooth recovery and was discharged with a Foley catheter, which was later removed following normal postoperative imaging results. Prompt diagnosis and intervention are crucial for managing pediatric BFBs to prevent complications. Imaging and cystoscopy play key roles in treatment.

## Introduction

Bladder foreign bodies (BFB) have been mentioned in the literature, with most cases reported in adult females. Rare cases have been noted in the pediatric population. BFBs can result from self-infliction, iatrogenic factors, migration from adjacent organs, or penetrating ballistic trauma [1]. Psychiatric disorders are the most common cause of BFB, followed by intoxication and erotic stimulation [2]. BFBs may enter the bladder through the urethra or by migrating from adjacent organs such as the vagina, rectum, or peritoneal cavity. The most common age group affected is boys aged 4.5 to 15 years and girls aged 10 months to 12.5 years [3]. Cystoscopy is typically the first approach in managing these cases, followed by laparotomy when endoscopic techniques are unsuitable or have failed [2, 3].

## Case presentation

We report the case of a 16-year-old female who presented to the emergency department with a [2-]day history of dysuria, painful defecation, and suprapubic pain, accompanied by nausea and anorexia. Upon examination, the patient was afebrile but exhibited tachypnea and tachycardia. Suprapubic tenderness was noted without abdominal distention. A rectal examination revealed a hard object palpated transversely ~6 cm from the anal verge. Initial abdominal X-ray demonstrated a transversely positioned foreign body in the lower abdomen. A subsequent non-contrast CT scan revealed an 8.2 cm longitudinal radiopaque foreign body traversing the left side of the pelvis, penetrating obliquely into the urinary bladder through the left postero-lateral wall.

**Figure 1:**
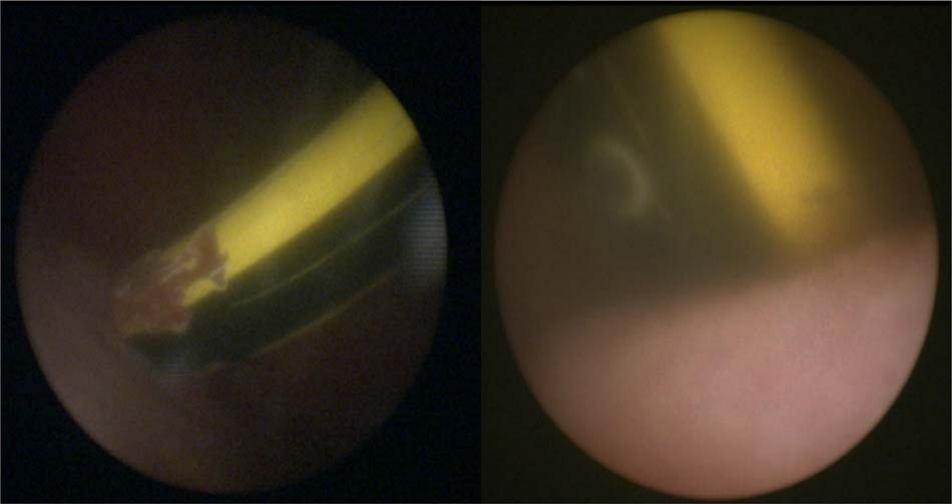
Cystoscopy showing the foreign body in the bladder.

**Figure 2:**
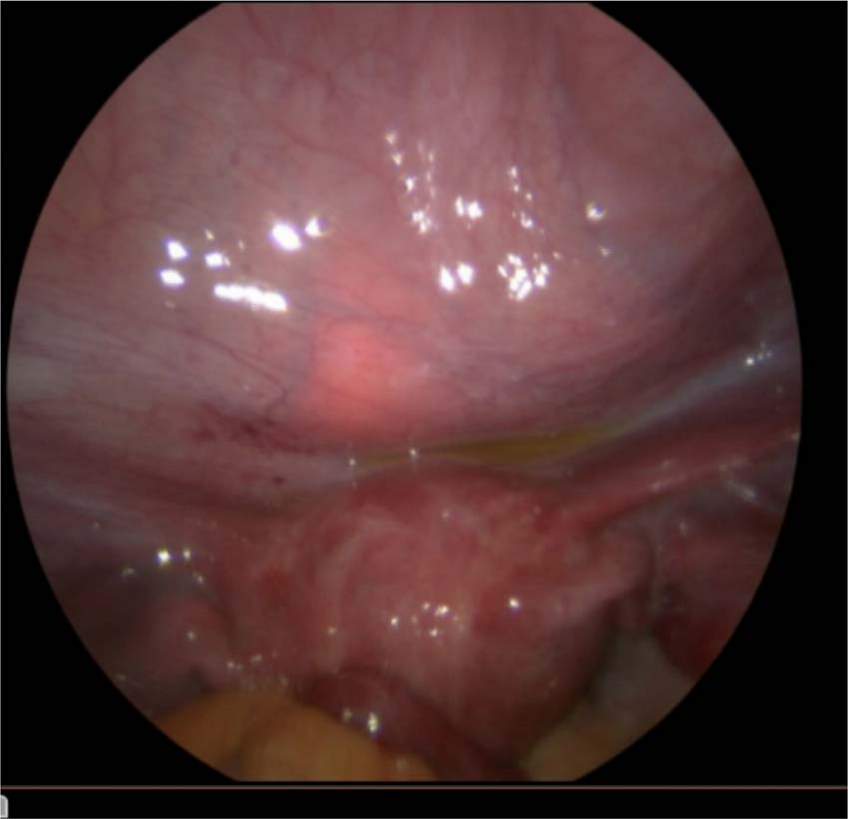
Diagnostic laparoscopy showing no bladder perforation.

**Figure 3:**
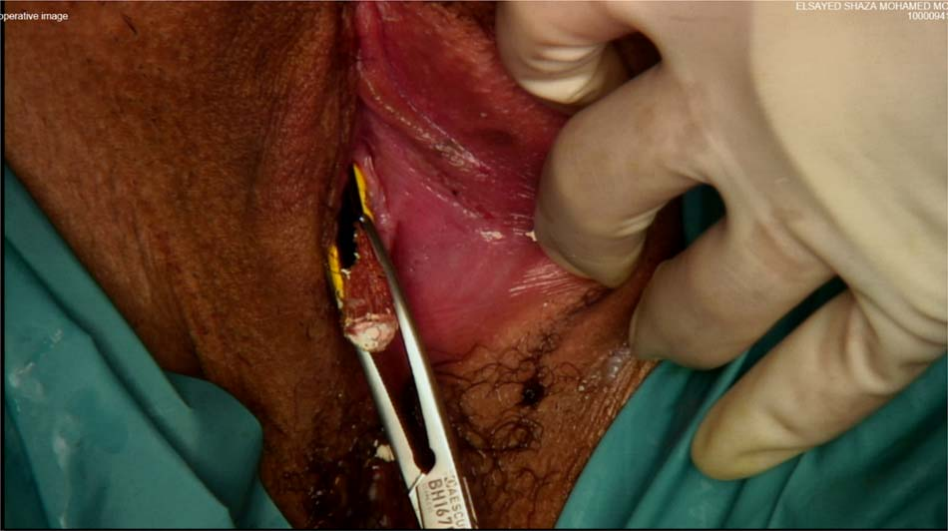
Forceps transurethral removal of the foreign body.

The patient was taken to the operating room for diagnostic laparoscopy, vaginoscopy, and cystoscopy. During laparoscopy (Fig. 2), minimal peritoneal fluid was observed, and no perforation was noted. Both the bladder and uterus appeared normal. Vaginoscopy findings were unremarkable. Cystoscopy (Fig. 1) revealed a normal urethra, with the lead pencil lying transversely within the bladder. The foreign body was successfully removed with forceps after multiple attempts (Fig. 3). Post-removal, a small injury site in the bladder wall was noted. The patient experienced a smooth postoperative recovery and was discharged with a Foley catheter after 2 days. Voiding urethrocystography conducted one week postoperatively indicated normal bladder capacity and contour, with no leaks, leading to the removal of the Foley catheter.

## Discussion

The presence of a foreign body in the bladder is considered rare in the pediatric population [3]. Most reported cases are due to self-insertion, iatrogenic factors, sexual abuse, perforation from adjacent organs, or traumatic routes [1, 4, 5]. The age and cause vary significantly between sexes [3]. In boys, the most common cause is self-insertion for autologous sexual stimulation during adolescence [3]. In girls, the presence of a lower urinary tract foreign body is often accidental at a very young age, with vaginal foreign bodies being more common than urethral ones [3]. Obtaining a history from patients, especially children and adolescents can be challenging and must be approached sensitively, as patients with self-insertion may feel shame. Consequently, patients often present to the emergency department later, driven by symptoms such as hematuria, urinary frequency, dysuria, abdominal pain, and occasionally urinary retention [4, 5]. Late presentations are common and can lead to complications for the urologist, including incrustation, stone formation, or migration through the bladder wall [5].

Diagnostic imaging plays a crucial role in the assessment and planning of the approach for these patients. While plain abdominal radiography and ultrasound can be used, CT scans are often necessary to determine the location, size, trajectory, and involvement of other organs of the foreign body before intervention [5].

The primary goal of treatment is the safe removal of the foreign body, typically achievable through the transurethral route, which is both a diagnostic and therapeutic approach. This technique is considered the first-line approach in most cases and can be combined with a laparoscopic approach [5]. In instances, where the endoscopic approach fails due to factors such as size, nature, position of the foreign body, surgeon experience, the presence of a sharp object, small urethra, or the availability of instruments, an open approach is pursued [4, 6]. Indications for proceeding with an open approach also include hemodynamically unstable patients, intraperitoneal perforation, or organ injury [5, 6].

Another approach mentioned in the literature is the use of a small suprapubic cystostomy guided by endoscopic visualization. This method was first used for the removal of a toy frog in a pediatric patient and was developed by urologists to remove large foreign bodies in a minimally invasive manner, allowing for quick recovery and decreased morbidity [7].

## Conclusions

Intravesical foreign bodies are not uncommon and can occur due to various mechanisms. Late presentation, particularly in self-insertion cases, is common and often associated with complications. Radiological imaging is essential to localize and determine the nature and size of these objects. Most cases reported in the literature were successfully managed via transurethral or transvesical approaches, depending on the specifics of each case. However, in some instances, open surgery is required.
